# Two‐dimensional‐NGC‐SENSE‐GRAPPA for fast, ghosting‐robust reconstruction of in‐plane and slice‐accelerated blipped‐CAIPI echo planar imaging

**DOI:** 10.1002/mrm.26179

**Published:** 2016-03-02

**Authors:** Peter J. Koopmans

**Affiliations:** ^1^ FMRIB CentreUniversity of OxfordOxfordUnited Kingdom.

**Keywords:** simultaneous multislice, multiband, CAIPI, Nyquist ghost, SENSE, GRAPPA

## Abstract

**Purpose:**

Ghosting‐robust reconstruction of blipped‐CAIPI echo planar imaging simultaneous multislice data with low computational load.

**Methods:**

To date, Slice‐GRAPPA, with “odd–even” kernels that improve ghosting performance, has been the framework of choice for such reconstructions due to its predecessor SENSE‐GRAPPA being deemed unsuitable for blipped‐CAIPI data. Modifications to SENSE‐GRAPPA are used to restore CAIPI compatibility and to make it robust against ghosting. Two implementations are tested, one where slices and in‐plane unaliasing are dealt in the same serial manner as in Slice‐GRAPPA [referred to as one‐dimensional (1D)‐NGC‐SENSE‐GRAPPA, where NGC stands for Nyquist Ghost Corrected] and one where both are unaliased in a single step (2D‐NGC‐SENSE‐GRAPPA), which is analytically and experimentally shown to be computationally cheaper.

**Results:**

The 1D‐NGC‐SENSE‐GRAPPA and odd‐even Slice‐GRAPPA perform identically, whereas 2D‐NGC‐SENSE‐GRAPPA shows reduced error propagation, less residual ghosting when reliable reference data were available. When the latter was not the case, error propagation was increased.

**Conclusion:**

Unlike Slice‐GRAPPA, SENSE‐GRAPPA operates fully within the GRAPPA framework, for which improved reconstructions (e.g., iterative, nonlinear) have been developed over the past decade. It could, therefore, bring benefit to the reconstruction of SMS data as an attractive alternative to Slice‐GRAPPA. Magn Reson Med 77:998–1009, 2017. © 2016 The Authors Magnetic Resonance in Medicine published by Wiley Periodicals, Inc. on behalf of International Society for Magnetic Resonance in Medicine. This is an open access article under the terms of the Creative Commons Attribution License, which permits use, distribution and reproduction in any medium, provided the original work is properly cited.

## INTRODUCTION

Simultaneous multislice (SMS) echo planar imaging (EPI) reconstruction algorithms suffer from interactions with EPI ghosting. These challenges and their interaction are introduced below, followed by a proposed solution.

### SMS Reconstruction

In SMS imaging, multiple slices are excited resulting in superimposed images that are unaliased using parallel imaging 1, 2, 3. Initial studies used SENSE‐GRAPPA 4 for reconstruction. Here, individual reference slices are concatenated along the phase encoding direction in image‐space. After transformation to k‐space, a conventional GRAPPA kernel 5 is estimated, effectively treating the N‐fold slice‐accelerated data as an N‐fold phase‐undersampled dataset of a concatenated image with an N‐fold larger field of view (FOV). In‐plane phase encode undersampling can be incorporated, i.e., both accelerations can be reconstructed with one kernel.

SMS image encoding can be enhanced with the CAIPIRINHA/CAIPI technique 6, 7, which alleviates the unaliasing problem and reduces g‐factor noise amplification by shifting the FOVs of individual slices with respect to one another in the phase encoding direction. However, the sharp phase and magnitude discontinuities that arise at the concatenation points of the shifted slices result in SENSE‐GRAPPA reconstruction artifacts. To avoid this, Slice‐GRAPPA 7 assigns a unique k‐space to each slice and, as opposed to SENSE‐GRAPPA, trains N *slice‐specific* kernels that project the SMS data onto each of these k‐spaces, while actively blocking signals from other slices: “Split‐Slice‐GRAPPA” 8. The downside is that Slice‐GRAPPA uses a separate reconstruction step to deal with any in‐plane acceleration after slice unaliasing because the projection kernels differ slightly from those used in the GRAPPA framework.

In SENSE‐GRAPPA slice‐interface artifacts can be avoided by extending the concatenated FOV in the phase encoding dimension with a FOV of zeros, resulting in a virtual acceleration factor of the SMS‐factor + 1 (not taking in‐plane acceleration into account) 9, 10, 11. Alternatively, one can concatenate along the *readout* dimension to avoid interaction with the CAIPI phase dimension 12. Reconstruction can then be performed serially (slice unaliasing in the readout direction followed by in‐plane unaliasing in the phase encoding dimension). Throughout this study this method will be referred to as 1D‐SENSE‐GRAPPA. A second variant is assessed that uses a single kernel to simultaneously reconstruct both dimensions, referred to as 2D‐SENSE‐GRAPPA. This study reports on Slice‐GRAPPA and 1D‐ and 2D SENSE‐GRAPPA methods in the context of EPI sequences that suffer from Nyquist ghosting, which can significantly hinder CAIPI‐SMS reconstruction.

### EPI/Nyquist Ghosting

Ghosting results from misalignment of positive and negative readout gradient lines due to, e.g., gradient/ADC delays, and eddy currents. Although ghosting can show significant nonlinear behavior 13, systematic differences between positive and negative lines are often approximated using linear corrections, shifting the lines based on reference scans 14, navigator lines 15 or by using reference‐free data‐driven methods 16. In SMS, these methods are not always applicable: different slices will typically exhibit different ghosting due to spatial dependence of the eddy currents. But, as these slices are aliased, they cannot be corrected differentially.

**Figure 1:**
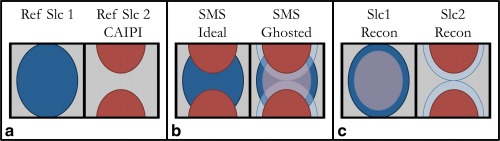
Nyquist ghosting interaction with CAIPI. **a:** Reference data for two slices, the second one being shifted by a CAIPI FOV/2 shift. **b:** Simultaneous acquisition of the slices depicted in panel a ideally results in the superimposed image on the left. Nyquist ghosting due to the EPI readout causes ghosts of each slice to “perfectly overlap” with the other slice's true location, effectively cancelling the controlled aliasing benefit for the ghosted signal. **c:** Reconstructing the ghosted data in panel b with the reference data of panel a will cause (part of) each ghost signal to be reconstructed as belonging to the other slice, and slice unaliasing will effectively have failed. The extent to which this occurs depends on how crucial CAIPI is w.r.t. the coil sensitivity profiles: the closer together the slices, the less resolving power is provided by the coils, hence the stronger this artifact becomes.

In initial SMS EPI work, only the average ghost was corrected before slice unaliasing, and afterward a slice‐specific correction was performed based on single‐slice reference scans 3. This was shown to be sub‐optimal as ghosting interferes with the slice reconstruction itself, particularly when used in combination with blipped‐CAIPI 17, especially when using a FOV/2 CAIPI shift. Ghosts are then prone to be assigned to a neighboring slice and subsequently slice‐specific ghost correction is no longer possible. This is illustrated in Figure 1.

This issue was solved by training two Slice‐GRAPPA kernels (odd and even), where these labels reflect the readout polarity of the first line of source points 17. This allows source lines to be shifted with respect to one another and the two kernels each reconstruct their own subset of k‐space lines separately, although not independently (as that would constitute doubling the undersampling factor). Because, in Slice‐GRAPPA, each slice has its own projection kernel, and odd–even kernels allow training on ghosted images, each kernel can be trained with the correct level of ghosting for that particular slice. These ghosts are removed after slice unaliasing resulting in clean EPI‐SMS reconstructions 17.

This report presents a method to perform odd–even reconstruction within the SENSE‐GRAPPA framework. Whenever the use of odd–even kernels is suggested, as opposed to SENSE‐GRAPPA in general, the prefix “NGC” (Nyquist Ghost Corrected) will be used. SENSE‐GRAPPA is attractive as it only uses GRAPPA operations and, therefore, is readily compatible with advanced reconstruction methods developed for GRAPPA [e.g., iterative 18, nonlinear 19, or sliding‐window 20 GRAPPA]. This includes the use of single‐step reconstructions with a 2D kernel 21, which is computationally favorable (see Appendix 1).

In 3D imaging, serial 1D and 2D reconstructions differed in terms of error propagation 21. Here, this is investigated in the context of SMS imaging. For 2D implementations of NGC‐SENSE‐GRAPPA, first an additional step is needed to make unaliasing of the in‐plane dimension robust to ghosting which is explained in the methods section.

## METHODS

**Figure 2:**
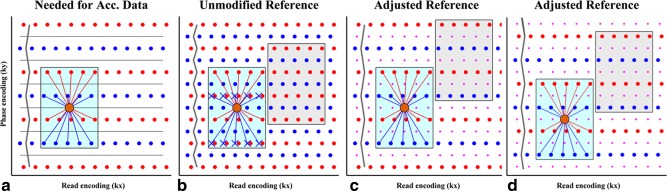
In‐plane kernel estimation in the presence of Nyquist ghosting. This figure shows how to preprocess reference data to obtain a kernel compatible with the ghosted, accelerated data. The procedure is depicted for a single slice but needs to be repeated for all slices before readout‐concatenation. Red and blue show k‐space samples that are shifted resulting in ghosting. Please note that any form of reference data could be used (here single shot EPI) as long as it can be made ghost‐free and the individual slice ghosting of the SMS data is known. **A:** The accelerated data together with an overlay of a 5x4 kernel. The lines indicate the source points that are needed to fill in the missing value at the location of the orange dot (please note that the coil dimension is omitted here for clarity). The gray line visualizes the frequency of the ghosting pattern. **B:** The reference data with the same kernel. Blue source points display a mismatch with the actual data. The cause is the higher frequency of the shifting pattern (grey zigzag line). The gray kernel outline indicates the next k_y_‐location for a kernel of this polarity (i.e., within the odd–even framework), shown translated in the read dimension for better visualization. **C:** The proposed fix to the reference data. The ghosting pattern in panel B is corrected and a new pattern is introduced that matches that of panel A. The changed periodicity causes the k_y_‐location of the next kernel (gray) to shift as well. This would reduce the number of equations in the inversion because, the purple samples are never used as source points. This can be avoided by introducing multiple shifting patterns, where the phase of the pattern is modified, and combining the sets of equations obtained before matrix inversion. **D:** A phase cycled variant of the zigzag pattern to increase the number of equations. The number of cycles required is equal to the in‐plane acceleration factor.

The interaction of in‐plane GRAPPA and ghosting is explained in Figure 2. In Figure 2a, the kernel is depicted that is needed to fill in the missing zero lines in the in‐plane accelerated data. The measured source points to be convolved with this kernel are shown in red and blue (indicating readout polarity), which are shifted with respect to the one another due to eddy currents etc. The target point (that is filled in) is indicated in orange and lies in the middle of the red and blue points, i.e., not shifted due to ghosting effects. Because the reference data (ACS lines; Figure 2b) were obtained without zero lines, the frequency of the red–blue shifting pattern has doubled (indicated by the grey zigzag line); thus, the kernel's source points do not line up with the measured points in the training data. This is a problem as one of the fundamental underpinnings of GRAPPA is that one has the ability to predict a missing point in k‐space based on a known multicoil relationship with its neighboring points. Ghosting or other physiological effects change this relationship and a mismatch of reference lines and accelerated data, therefore, is a general problem in parallel imaging, not specific to SMS reconstruction 22.

However, in non‐SMS or serial 1D SMS, reconstruction ghosting in particular is not a severe issue as one simply corrects both the ACS and the imaging data before in‐plane unaliasing. In single‐step 2D reconstruction, this is not possible as the SMS data cannot be made ghost‐free due to the variability of the ghosts of the underlying slices.

The proposed solution is to alter the reference data to have the same pattern as the accelerated data. This requires the ghosting parameters of the individual slices underlying the SMS data to be known (as is the case in Slice‐GRAPPA and 1D‐SENSE‐GRAPPA). By appropriately shifting the lines of the reference scan the correct pattern can be created (Fig. 2c). Once each reference slice is “corrupted” with its individual line‐shifting pattern, the slices are concatenated in the readout direction and a 2D‐GRAPPA kernel can be estimated. As these kernels are now trained on ghosted data, the odd‐even strategy *must* be used.

Figure 2c also shows that the introduced shift pattern does reduce the number of positions that the kernels can assume in the estimation process, decreasing the number of equations in the kernel inversion: The points indicated in purple are neither on the correct location to serve as source points for the odd‐labeled kernel, nor for the even one, but in fact line up with the target point in orange, such that after unaliasing the pattern can easily be removed. This loss can be avoided by repeating the process with a different zigzag pattern with as shown in Figure 2d. The number of such phase cycles needed is equal to the in‐plane acceleration factor and the same number of equations is obtained as would have been with conventional methods.

Please note that, in this example, single‐shot EPI reference data are assumed. Had segmented data been used (to match distortions), the reference data would have been two red lines being followed by two blue lines etc., whereas using a GRE scan would have resulted in nonshifted lines. In general, any discrepancy between the ghosting pattern of the reference and the imaging data can be addressed in a similar manner as long as (i) the reference data can be made ghost free, and (ii) the ghosting parameters (zigzags) of each of the slices underlying the SMS imaging data are known.

**Figure 3:**
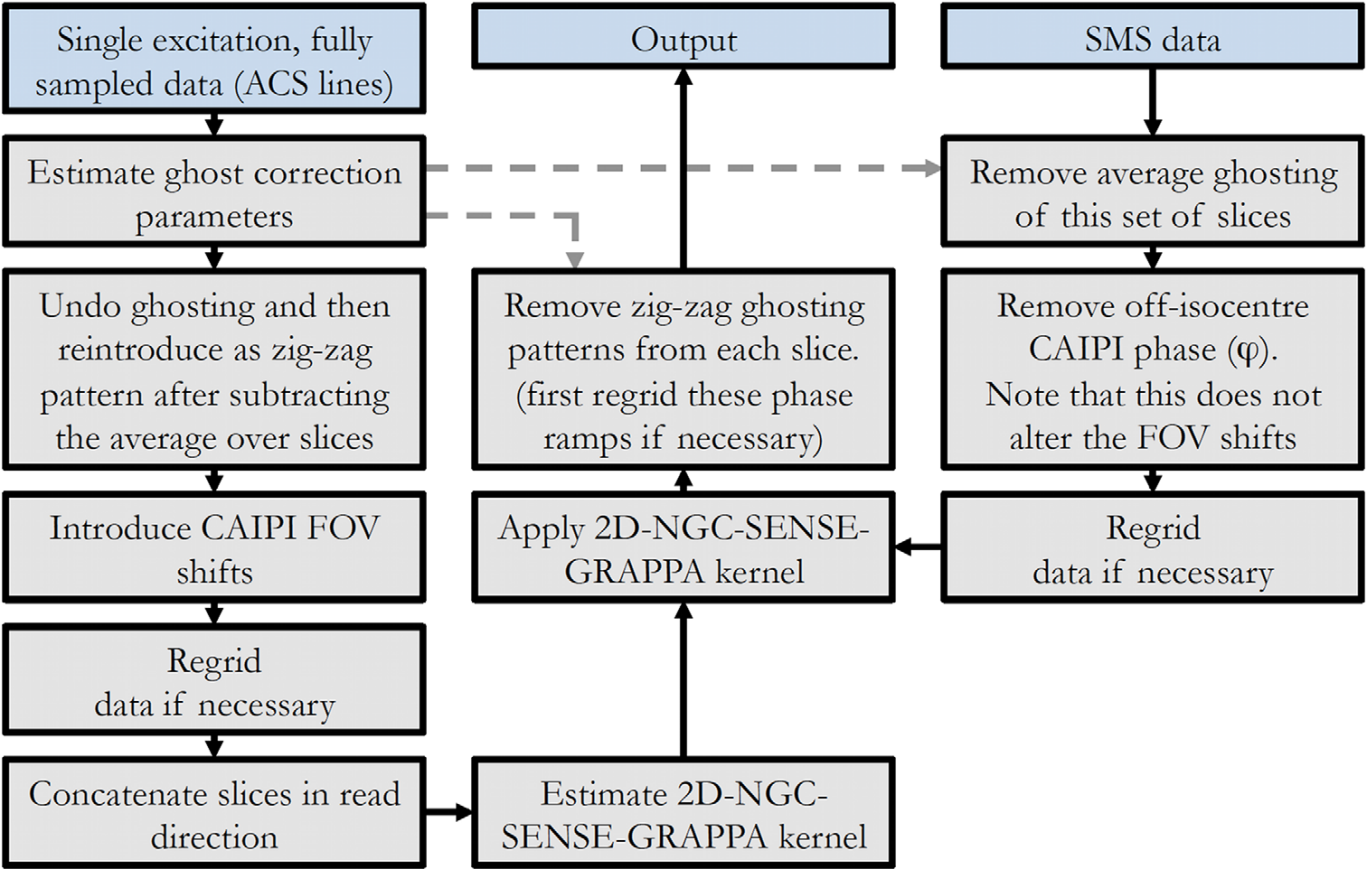
Reconstruction pipeline for 2D‐NGC‐SENSE‐GRAPPA.

After 2D unaliasing, the introduced shifting patterns are removed from the slices to form ghost‐free images without enhanced residual aliasing that would otherwise be present due to the interaction of the Nyquist ghosting, in‐plane acceleration, and the blipped‐CAIPI FOV shifts. The entire reconstruction pipeline is shown in Figure 3.

### Experiments

Reconstructions were performed with SENSE‐GRAPPA (1D and 2D) and Slice‐GRAPPA, including the leakage blocking modification 8. For 2D‐NGC‐SENSE‐GRAPPA the pipeline in Figure 3 was followed. For Slice‐GRAPPA and 1D‐NGC‐SENSE‐GRAPPA, the SMS data were first average‐ghost corrected. After slice unaliasing, residual ghosting in the individual slices was addressed before in‐plane GRAPPA reconstruction.

The three reconstruction methods were tested on gold‐standard simulated SMS data where known ghosting was introduced in multiple scenarios: (i) odd‐even kernels versus their normal variants, and the influence of kernel sizes on quality and computation time; (ii) local phase modulation simulating breathing after kernel estimation; and (iii) rigid body motion after kernel estimation. Real SMS EPI datasets were subsequently reconstructed and the fidelity was assessed visually due to the lack of a gold standard.

All methods were coded in MATLAB's standard m‐file format without use of precompiled MEX modules and executed on the same computer (2.6 GHz Intel Core i7 quadcore, 16 GB RAM), which is particularly relevant for the computation time comparisons.

### Gold Standard Simulation Experiments

Simulated SMS data were generated from an MP‐RAGE scan (24‐year‐old male after informed consent, 3 Tesla (T) (Siemens Verio, Erlangen, Germany), 32‐channel headcoil), to serve as a gold standard without ghosting. No acceleration was used to avoid interaction with the assessed methods. Relevant sequence parameters were: field of view (FOV) 256 × 256 × 216 mm^3^, matrix 192 × 192 × 144, echo time/inversion time/repetition time (TE/TI/TR): 5.7/900/2000 ms, right–left phase encoding. Posthoc, to closer resemble typical EPI brain acquisitions, the FOV was cropped to 228 mm and data were downsampled to a 120 × 120 matrix, resulting in 1.9 mm in‐plane resolution.

Three slices were taken with a spacing of 15 slices (22.5 mm), the odd phase encode lines of the slices were shifted by ‐0.75, ‐0.5, and +0.5 k‐space units, respectively, well within range of typical EPI observations and chosen such that, after subtraction of the average, none would be zero.

The ghosted data served as single‐slice reference data. SMS data were created by summing two‐fold in‐plane undersampled copies of these slices after applying a CAIPI shift to the middle slice of half an acceleration‐reduced FOV.

### Assessment of Reconstruction Fidelity

The difference between the original and reconstructed slices was expressed relative to the average signal intensity of the brain as opposed to voxelwise division of the two images to avoid inflating error measures in areas of low intensity. The difference images were spatially smoothed (Gaussian, full width half maximum = 15 mm) to suppress high‐frequency g‐noise, which is not ghosting‐related and not of interest here. Also, only voxels inside the brain were taken into account: even if a reconstructed ghost is masked out, its signal is still missing from the original image, thereby adding to the error while this mask effectively excludes the contribution of rectified noise in out‐of‐brain voxels, which would cause a reconstruction‐independent “offset” in the error measures. To aid interpretation, the figures are accompanied by histograms over all brain pixels. Narrower histograms indicate better reconstructions. The mask was created with FSL‐BET 23.

### Kernel Geometries

**Figure 4:**
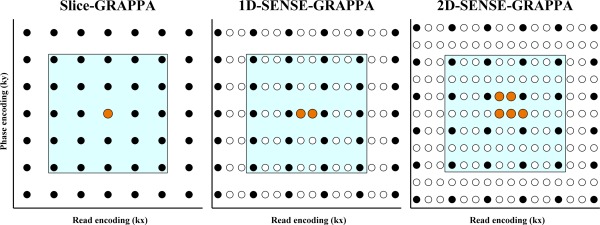
Slice unaliasing kernel geometries. Due to implementation‐specific differences, the kernels used in this study vary in geometry. As a rule, whenever data are fully sampled in a dimension, that dimension's kernel size is odd, otherwise it is even. The examples shown all use three‐fold slice‐acceleration, the black dots indicate source points, the orange ones targets, and white circles are missing values. As 2D‐SENSE‐GRAPPA deals with in‐plane acceleration at the same time, its figure shows two‐fold undersampling as well. The other two methods consider the phase encode dimension to be fully sampled as in‐plane acceleration is dealt with at a later stage. In the Appendix, kernel sizes are indicated with K1 and K2, and the different accelerations with AF1 and AF2. For the 2D‐SENSE‐GRAPPA case these would have the following values: K1 = 4 (four source points in the read‐direction), K2 = 4 (four source points in the phase‐direction), AF1 = 3 (two missing lines in the read‐direction, which doubles as the slice‐direction in this algorithm), and AF2 =2 (1 missing line in the phase‐direction).

The methods were all implemented using kernels in which the source points were symmetrically distributed around the target points. Whenever a sampling pattern has a missing value due to acceleration in a particular dimension, a kernel will have an even number of points in that dimension. In fully sampled dimensions, the kernel will have an odd number of points. Please note that this is unrelated to the odd–even kernel distinction mentioned earlier. Graphical depictions are shown in Figure 4.

### Computation Times

One‐step reconstructions are more computationally efficient than two serial steps. This is shown analytically in Appendix 1 and predicted calculation times for the odd–even kernel estimation are plotted on top of the measured values. To reduce the influence of practical code implementations when measuring the computation time, only the durations of the two most important computation steps were taken into account: (i) The matrix inversion that estimates the kernel from the large set of equations in the kernel estimation phase. (ii) The loop that applies the kernel to each accelerated data volume. In all methods the loop structures were very similar (k‐space convolution), as were the variable initializations that happened inside the measured timing window, hence coding efficiencies were approximately equal.

### Simulation of Breathing Artifacts

One‐step and two‐step reconstructions are expected to behave differently in terms of error propagation 21. On the one hand, two‐step approaches allow corrections to be applied at the intermediate stage (like ghost corrections of unaliased slices as performed in this study), on the other hand, if the slice unaliasing step underperforms for any reason, these errors can introduce additional problems during the in‐plane reconstruction stage (e.g., ghost correction applied to signal originating from a different slice). Scenario 2 simulates propagation of breathing errors: the kernel is estimated in a fully exhaled state and subsequently applied to data in a fully inhaled condition.

Not only does breathing cause bulk motion (assessed in scenario 3), but it also changes the resonance frequency, spatially varying across the brain. The frequency difference between inhale and exhale is 2–8 Hz at 7T 24, 25. Assuming TE = 30 ms for a functional MRI (fMRI) EPI scan, 8 Hz would lead to a phase change of 86°. To mimic spatial variation of the field, the phase modulation was implemented as a 2D Gaussian with 86° as its maximum amplitude and a FWHM of half the simulated FOV 24, 25. The frequency modulation strongly depends on the distance to the lung and the rate of decay of this frequency when moving away from the lungs is 0.1 cm^‐1^ 25. With the simulated slice spacing of 2.25 cm, this means that if the bottom slice has an offset of 8 Hz, the next simulated slice will have an offset of 6.2 Hz. The modulations were applied to the SMS data before the CAIPI manipulation.

### Simulation of Motion Artifacts

Similar to the breathing scenario, motion error propagation was assessed. After kernel estimation, image data were rotated (before SMS summation) by 0.25° around the z‐axis followed by 0.25° around the y‐axis. Subsequently, the brain was translated by 0.5 mm in all three cardinal directions. These values are well within the range of motion typically observed in fMRI of healthy persons.

### Reconstruction of Real SMS Data

#### Ghost Correction of Real EPI Data

The algorithms all require knowledge of slice‐specific ghosting parameters, which were unknown for the nonsimulated data. A data‐driven approach was used similar to 16: for each slice, MATLAB's “fminsearch.m” determined two coefficients defining a linear phase gradient that, when applied to the Fourier transform of the negative readout gradient lines in k‐space, maximizes the sum‐of‐squares of the image intensity values. Local minima were avoided by using 210 initializations.

#### Scenario 1: Conventional SMS‐EPI Diffusion Imaging

Odd‐even Slice‐GRAPPA and 2D‐NGC‐SENSE‐GRAPPA were used to reconstruct data from an SMS diffusion sequence distributed by the HCP consortium 26, 27, 28. Some modifications were made to the HCP protocol to make the data compatible with 2D‐SENSE‐GRAPPA due to different requirements on the reference data (see the Discussion section); therefore, no comparisons to the HCP data should be made. Four sequence variations were assessed.

Parameters were: SMS factor 2 or 3, in‐plane acceleration factor 2, CAIPI FOV/3 shifting, FOV 192 mm, matrix 160, 104(52) or 120(40) slices, slice thickness = 1.2 mm, TE = 70 ms, EPI echo spacing 0.76 ms, 6/8 partial Fourier. Next to nonaccelerated reference data (ACS lines), single‐slice, in‐plane accelerated images (indicated here with “REF2”) were acquired for use in Slice‐GRAPPA. Two variants of ACS data were acquired: single‐shot with 38 phase‐encoding lines, and segmented acquisition with 76 lines. The latter had identical geometric distortion as the imaging data but this comes at the cost of increased phase instabilities (unfortunately, a FLEET‐like solution 22 was unavailable). To minimize phase inconsistencies, each shot of the segmented ACS data was first EPI‐ghost corrected using the method described above. The two segments were subsequently merged and the same correction algorithm was run again, this time estimating shot‐to‐shot phase fluctuations. This markedly reduced segmentation ghosting although results were not perfect due to the nonlinear nature of the physiologically induced phase differences.

The Slice‐GRAPPA reconstruction pipeline was as follows: average ghost removal, slice‐unaliasing using REF2 data, single‐slice ghost correction, in‐plane unaliasing using ACS. The 2D‐NGC‐SENSE‐GRAPPA algorithm only used the ACS data.

#### Scenario 2: Read‐Out Segmented Diffusion Imaging

In the scenarios above, the ACS data were either not distortion matched or suffered from intersegment phase instabilities, which disadvantaged 2D‐SENSE‐GRAPPA (see the Discussion section). In Scenario 2, a readout‐segmented sequence was used: this sequence is designed to minimize distortions 29 and, therefore, the distortion mismatch was expected to have less impact.

Spin‐echo EPI data were acquired at 7T (Siemens, Erlangen, Germany) using an SMS version of readout‐segmented EPI with sinusoidal readouts 30. Parameters were: SMS factor 3, in‐plane acceleration factor 2, FOV 210 mm, matrix 140, 81 27 slices of 1.5 mm, TE = 60 ms, echo spacing 0.36 ms, 32 channels, CAIPI FOV/2.

Initial reconstructions with non–odd–even Split‐Slice‐GRAPPA proved troublesome with ghosting clearly interacting with CAIPI, particularly for the bright cerebrospinal fluid (CSF) signal. To demonstrate the improvement offered by odd–even kernels, the results section also contains this flawed reconstruction. Odd–even kernels did not resolve all artifacts, however, which motivated the development of the alternative NGC‐SENSE‐GRAPPA methods described in this study.

All methods were applied with different kernel sizes in the range of 4 to 8, and for each method, its best reconstruction was determined visually and used in the comparison.

## RESULTS

### Simulated Scenarios

#### Scenario 1: The Effect of Kernel Size and the Use of Odd–Even versus Normal Kernels

**Figure 5:**
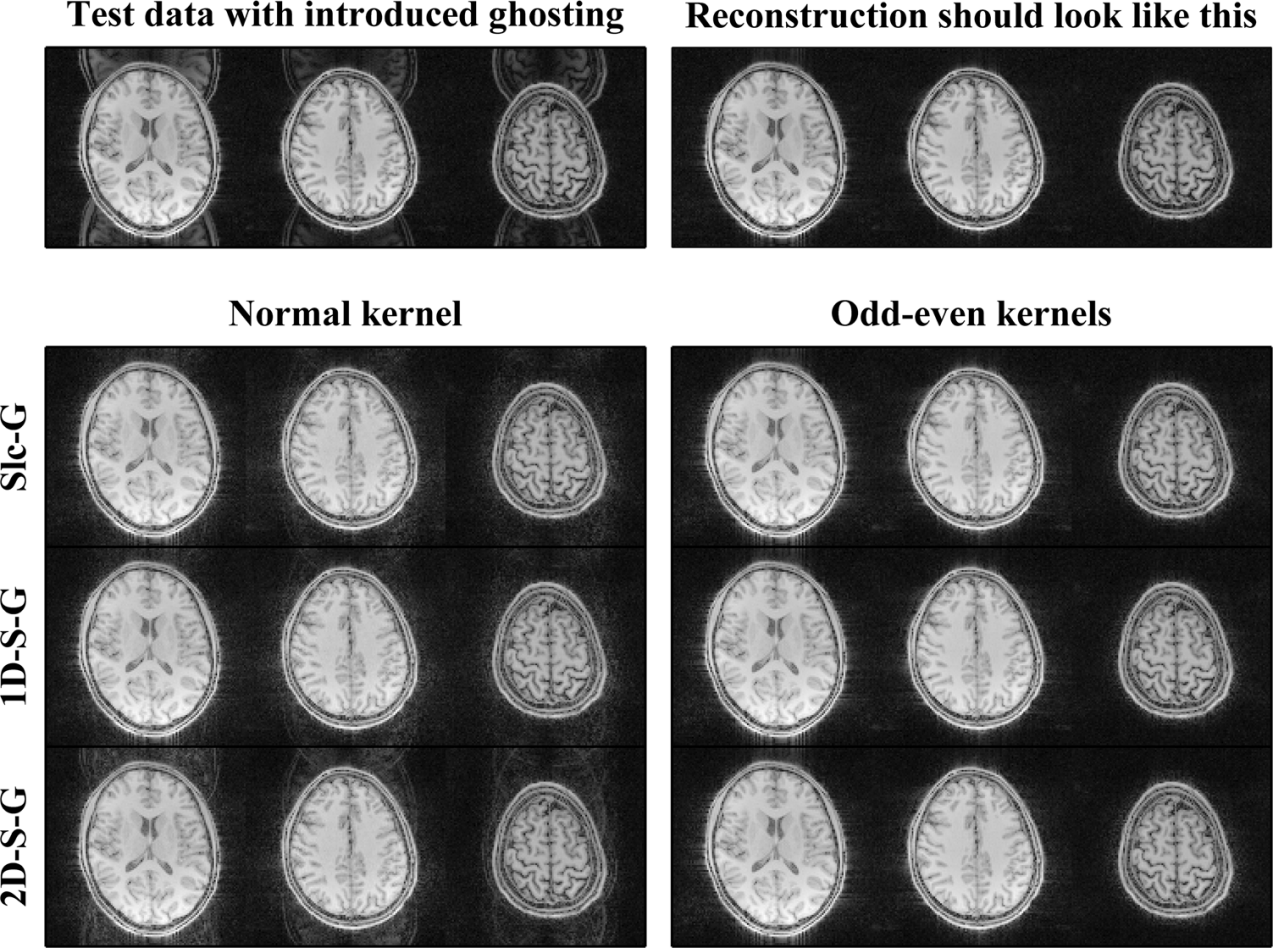
Reconstruction example of the gold standard dataset. The top row shows the data after introducing the ghosting parameters as described in the methods on the left, and the unaltered data on the right. The bottom panel on the left shows reconstructions without the use of odd–even kernels (and in the case of 2D‐SENSE‐GRAPPA without the in‐plane ghost correction described in Figure 2). On the right, the reconstructions with ghost‐correcting kernels are shown. In these examples, kernel sizes were: 7 × 7 for Slice‐GRAPPA, 7 × 6 for 1D‐SENSE‐GRAPPA, and 6 × 6 for 2D‐SENSE‐GRAPPA.

**Figure 6:**
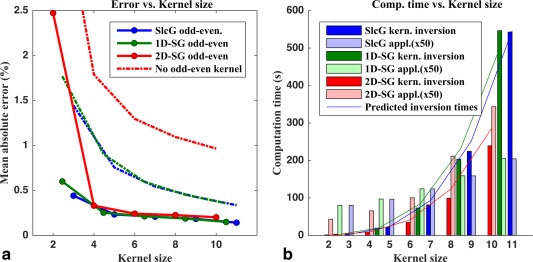
Reconstruction errors and computation time versus kernel size. The x‐axes denote kernel sizes expressed as the square root of the number of points used in the slice unaliasing kernel (i.e., noninteger for the 1D‐SENSE‐GRAPPA method, which uses rectangular kernels, see Figure 4). For both Slice‐GRAPPA and 1D‐SENSE‐GRAPPA, the follow‐up in‐plane kernel scales along with the slice unaliasing one as [N × N‐1]. In all cases, the SMS factor was 3 and the in‐plane acceleration factor was 2. **A:** The reconstruction error within the brain mask is shown expressed as the average absolute value of the relative error w.r.t. the gold standard. The dashed lines belong to reconstructions where the odd–even method was not applied. This results in large ghosting artifacts (see also Figure 5). The 2D‐SENSE‐GRAPPA performs worst here because the other two methods still attempt to correct part of the ghosting before in‐plane unaliasing whereas this cannot be done in the 2D case. When using the odd–even method the three methods perform very similar with only a very slight disadvantage to the 2D method. Slice‐GRAPPA and 1D‐SENSE‐GRAPPA perform identically. **B:** The computation times for the three odd–even methods are shown. The solid colors indicate the time it took for the matrix inversion in the kernel estimation stage (2 inversions for odd–even slice unaliasing, plus 3 for in‐plane unaliasing in the 1D methods; two larger inversions for odd–even 2D‐SENSE‐GRAPPA). The solid lines show the predicted computation time based on the computational complexity of the pseudoinverse. The lighter colors indicate the application times for 50 measurements (putting them on a similar scale). In all cases the 2D‐kernel inversion is faster than the other methods. On the application side, 2D‐SENSE‐GRAPPA is faster than the other two for kernel sizes below 8.

Simulation data were reconstructed with different kernel sizes (example reconstruction in Figure 5), the reconstruction error versus kernel size is shown in Figure 6A. Errors behaved very linearly provided the kernels were large enough (not poorly determined). Computation times are shown in Figure 6B along with their predicted values (see Appendix 1).

#### Scenario 2: Simulated Breathing Artifacts

**Figure 7:**
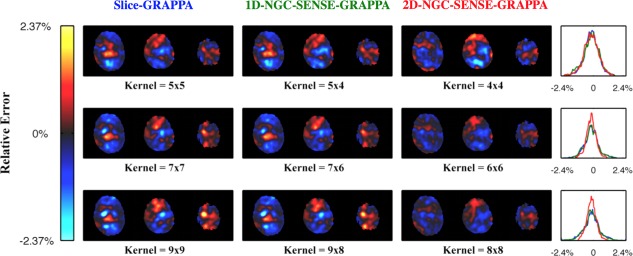
Simulation of error due to breathing. Rows show different kernel sizes. Columns indicate the methods used, the title colors corresponding to the error histograms in the last column. Slice‐GRAPPA and 1D‐NGC‐SENSE‐GRAPPA perform identically. For kernel sizes larger than four, 2D‐NGC‐SENSE‐GRAPPA outperforms the other two methods leading to narrower error histograms.

Figure 7 shows that both serial 1D methods show larger reconstruction errors than the 2D variant. Other values for the width of the Gaussian and the amplitude were tested too, but this affected the results to a limited extent without changing the overall picture. Leaving out the z‐dependence of the frequency modulation also made little difference, suggesting that it is mainly the within‐slice, nonlinear mismatch of the ACS data that drives the error, not the variation of this mismatch between slices.

#### Scenario 3: Simulated Motion Artifacts

**Figure 8:**
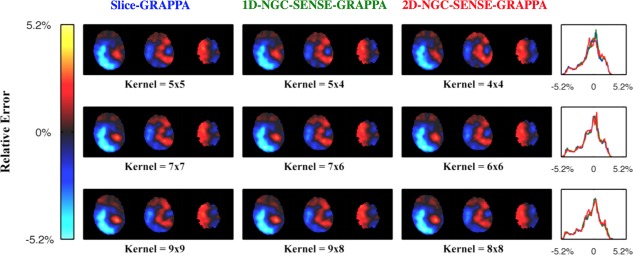
Simulation of error due to motion. Rows show different kernel sizes. Columns indicate the methods used, the title colors corresponding to the error histograms in the last column. The three methods perform equally and increasing kernel sizes does not have an effect on the error.

Results of scenario 3 are shown in Figure 8 with no difference between the methods over a range of kernel sizes.

#### Real SMS Data, Single‐Shot EPI

**Figure 9:**
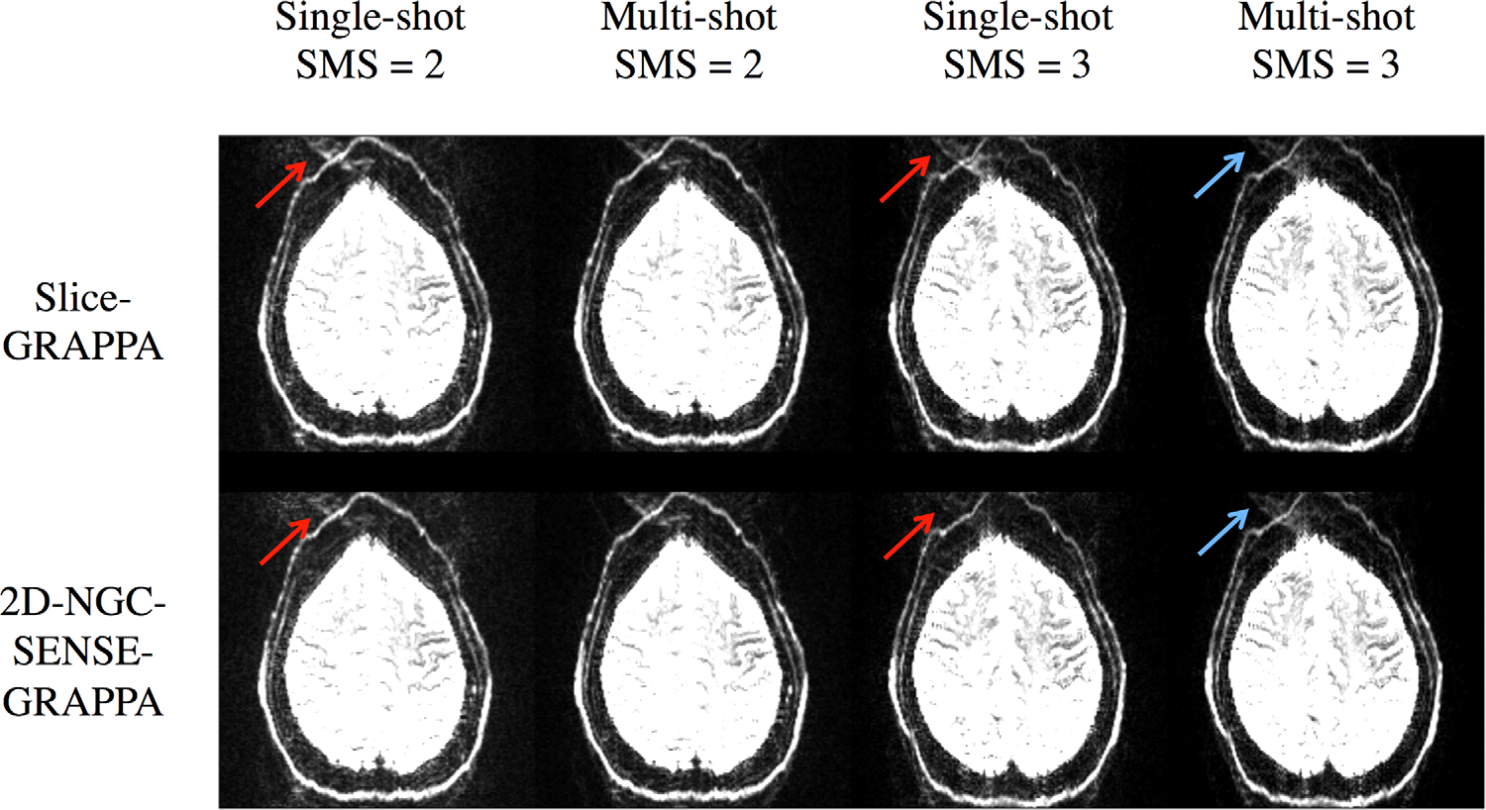
Reconstructions of SMS EPI data using the CMRR distributed diffusion sequence. Rows show Slice‐GRAPPA (7 × 7 kernel) and 2D‐NGC‐SENSE‐GRAPPA (6 × 6) reconstructions of four different scans of the same subject, slices are approximately matched to show the same location and are scaled identically. For single‐shot acquisitions, 2D‐NGC‐SENSE‐GRAPPA shows slightly less ghosting as the Slice‐GRAPPA reconstruction (red arrows). In the case of segmented ACS lines (multishot) and an SMS factor of 3, the Slice‐GRAPPA method performs slightly better (blue arrows).

The results for the four scenarios are shown in Figure 9 where 2D‐NGC‐SENSE‐GRAPPA performs similar to Slice‐GRAPPA. The biggest difference can be seen in the multishot ACS scenarios where Slice‐GRAPPA performs slightly better than 2D‐NGC‐SENSE‐GRAPPA (blue arrows). This is likely due to phase‐corruption in the segmented ACS data. The 2D method uses these for both slice and in‐plane unaliasing, whereas the Slice‐GRAPPA algorithm could take advantage of the other reference data, REF2 which was not segmented. In the single‐shot ACS scenarios, 2D‐NGC‐SENSE‐GRAPPA may perform slightly better (red arrows) but some residual ghosting is still present.

#### Real SMS Data, Readout‐Segmented EPI

**Figure 10:**
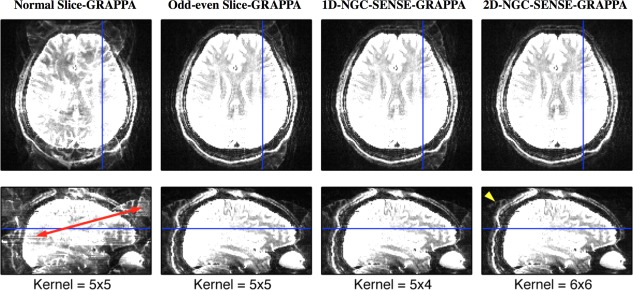
Reconstructions of readout‐segmented SMS EPI data. The blue lines indicate the positions of the cuts shown. Kernels used are indicated on the lower row. Image scaling was identical in all images and set to 10% of the maximum image value. Slice‐GRAPPA without odd–even kernels clearly shows leakage of another slice's ghost signal (indicated by red arrow). Odd–even Slice‐GRAPPA and the equivalently performing 1D‐NGC‐SENSE‐GRAPPA both massively improve the reconstruction, but nevertheless show residual four‐fold ghosting. Such ghosting occurs when in‐plane unaliasing is applied to ghosted data, indicating the ghost correction after slice‐unaliasing has not worked properly. When using 2D‐NGC‐SENSE‐GRAPPA the majority of this artifact disappears although at the top of the brain a small CSF ghost remains (yellow arrowhead).

Sagittal and transversal cuts through the reconstructed volumes are shown in Figure 10, all narrowly windowed (maximum display intensity equals 10% of the maximum voxel value) such that the ghosting artifacts are clearly visible. Whereas Slice‐GRAPPA and 1D‐NGC‐SENSE‐GRAPPA still show ghosting, 2D‐NGC‐SENSE‐GRAPPA shows a much better result.

## DISCUSSION

### Speed

Blaimer et al presented two methods for reconstructing data that were undersampled in two dimensions, the 2D‐GRAPPA method and the 2D‐GRAPPA‐*operator* method, the latter referring to two serially executed 1D kernels 21. Serial reconstruction was slower due to the requirement of two separate inversions, as shown in the Appendix. The computational burden, however, is dependent on the specific algorithm that is used to calculate the pseudoinverse. For other implementations, similar calculations would need to be made to decide which method is faster.

Figure 6B confirms that 2D‐SENSE‐GRAPPA is computationally less expensive than the serial methods. Matrix inversion was always faster, regardless the kernel size and in the kernel application stage the advantage was lost only for extremely large kernels. It should be noted, however, that the applications were performed using convolution in k‐space, whereas one can also use matrix multiplication in image space 31. This can reduce the computational burden at the cost of memory requirement and for such applications similar efficiency calculations would need to be done.

### Quality

Blaimer et al showed that serial methods yielded slightly higher image quality because kernels were individually better determined (illustrated in Figure 4 where the 2D kernel has a less favorable ratio of blue versus orange dots). The authors did warn about error propagation, however, and proposed to use a principle component analysis to determine which dimension should be unaliased first. In this study, these issues were examined in the context of SMS imaging where the quality of the reference data proves to play a vital role.

Figure 6A shows that in gold‐standard simulations without propagation errors 2D‐NGC‐SENSE‐GRAPPA has a slightly larger error indicating that indeed two‐step approaches are better determined. Slice‐GRAPPA or 1D‐NGC‐SENSE‐GRAPPA did not show any differences in any of the scenarios tested in this study, which is noteworthy considering the differences in theory underlying both methods.

As soon as the possibility of error propagation was introduced differences between serial 1D and 2D reconstructions could be seen. The breathing simulation clearly showed that the 2D method was able to achieve higher reconstruction fidelity. In the single‐shot EPI experiments, however, it proved to be better to first unalias the SMS slices based on an in‐plane accelerated single‐slice acquisition (i.e., REF2 with matched distortion) than to use the fully sampled reference ACS data to unalias everything in one go. Finally, when the distortion problem was alleviated using an intrinsically low‐distortion readout‐segmented EPI sequence better reconstructions were obtained with the single‐step 2D algorithm.

In the rigid body motion simulation, all methods suffered equally. A possible explanation is that unlike in the other tests where nonlinear differences between reference and imaging data were introduced, in the motion case, it are the coil sensitivities themselves that are wrong which affects all methods equally and linearly.

Reconstructions of readout‐segmented EPI SMS data clearly confirm the benefit of the use of odd–even kernels 17. Although both serial 1D‐reconstructions do not show slices with obvious components from other slices following the mechanism in Figure 1, they do both contain a residual four‐fold ghosting pattern. Such artifacts occur when two‐fold in‐plane accelerated data containing line‐to‐line inconsistencies are unaliased with kernels that were not trained on this pattern. Residual ghosting in Figure 10 is likely caused by the slice‐unaliasing step failing to exactly reproduce the original ghosts in each individual slice (through for example the filtering effects of the GRAPPA operation or because the kernel fit fails to capture the ghosting pattern). Therefore, afterward, the reference‐data‐based ghost correction before in‐plane reconstruction fails, resulting in poor in‐plane reconstruction.

Attempts to improve ghost correction by abandoning reference data and directly estimating ghosting using the data‐driven approach did not resolve this issue. In other words, the slice‐unaliasing step has introduced or left an inconsistency between odd and even lines in the data that cannot be corrected by applying linear phase ramps along the readout.

An alternative explanation would be that the ghosts in the reference data did not match those in the SMS data, as this experiment is not as well controlled as the simulations. However, as 2D‐NGC‐SENSE‐GRAPPA was able to reconstruct the images in Figure 10 without four‐fold ghosting this seems an unlikely candidate explanation. The results are in line with the differences in sensitivity to error propagation as demonstrated in the breathing simulation: even though the cause of the residual ghosting after slice‐unaliasing is unknown, the 2D reconstruction is more robust against it.

### Ghosting Robustness

Gold‐standard simulations allowed testing of the algorithms in the presence of large sets of perfect ACS lines. The method was tested on simulated SMS data into which known linear ghosting was introduced. This is a simplification of true EPI data, where nonlinear or nonstationary ghosts can occur as well. Nonlinear ghosts are not necessarily a problem; however, as long as they can be measured and corrected, the flow chart in Figure 3 remains appropriate. Please note that the requirement to be able to measure and correct the ghosting is shared by all methods. A nonlinear effect appears in the top 6 mm of the brain in Figure 10 where CSF signal still shows residual ghosting. The presence of nonlinearity was confirmed by investigating a nonphase encoded reference scan.

Nonstationary ghosts are an unresolved issue. Two types can be distinguished (slow/fast). If the ghosting parameters vary significantly over the time series due to, e.g., cryostat heating, the reconstruction quality of later volumes in the dataset could suffer. This, however, is true for all reconstruction methods presented here: if ghosting levels change, kernels should be re‐evaluated which for an entire image series would take relatively long. If one were to do it, however, the new ghosting parameter would be needed for each slice individually. A possible solution could be to use the time course of ghosting estimates of aliased SMS data as an indicator of the change in individual slices under the approximation that they all scale similarly.

A case of fast nonstationarity would occur if there was significant variation throughout the echo train. This is potentially more harmful as it violates one of the fundamental underpinnings of GRAPPA: the kernel should be translationally invariant. This would, therefore, preclude kernels in any of the methods to be evaluated on ghosted data, and probably a generalization of the odd–even kernel method allowing even more kernels would be needed to solve this but this is beyond the scope of this study. Investigation of nonphase encoded reference scans revealed that in the protocols presented here the effects were very stationary.

Finally, spatially varying ghosting in SMS acquisitions can partially be addressed by applying the ghost correction on a coil‐wise basis. This ensures that the correction is dominated by the slice that is closest to the coil to alleviate the problem to an extent 27.

### Interpretation

This study shows that using the odd–even kernel strategy and readout‐concatenation allows 1D‐NGC‐SENSE‐GRAPPA to perform the same as Split‐Slice‐GRAPPA under all circumstances explored here. The choice between the two will, therefore, mainly depend on practical considerations: advanced methods have been developed within the GRAPPA framework 18, 19, 20 readily compatible with SENSE‐GRAPPA and could, therefore, be exploited with minimal effort. On the other hand, large‐scale SMS projects such as the HCP 27 have adopted Slice‐GRAPPA and have brought online scanner implementations to a high level of maturity, and these can easily be used for other EPI sequences.

The overall picture of the results is that single‐step 2D algorithms may be able to perform very well but that acquiring the best possible ACS data is key to obtaining high‐quality reconstructions. The importance of good reference data is a topic of active investigation, and specifically the FLEET‐like ACS data acquisitions could offer a good solution 22. These allow distortion‐matched ACS lines to be acquired using a segmented approach while alleviating the effects of intersegment physiological fluctuations that would otherwise corrupt the ACS data.

The goal of the study was to obtain reconstructions with minimal residual ghosting and compare them to ones obtained with established techniques, similar to the ones used in the HCP project in this case. It is important, however, to stress that reported differences should not be over‐interpreted. First of all, the HCP protocols are highly optimized, but their reference data acquisition was incompatible with the 2D algorithm tested here. Therefore, the protocols needed to be adjusted, which precludes a direct comparison. Furthermore, it is incredibly difficult to oversee the impact the changes in residual ghosting have on elucidating biological information. Metrics of interest in large‐scale studies like the HCP (e.g., tractography results) are not easily re‐evaluated using a wide range of reconstruction algorithms (or any other preprocessing step for that matter) and, therefore, the results of this study should not be interpreted at that level.

Although treated in this study in the context of SMS imaging where the issue of ghosting differences between excited locations is quite apparent, one could argue that a similar issue would occur in 3D‐EPI 32. Given the equivalence of 3D‐CAIPI‐EPI and blipped‐CAIPI SMS EPI reconstructions 11, 33, it may prove advantageous to implement a similar ACS modification scheme as shown in Figure 2 together with the odd–even kernel estimation in 3D imaging. By using a 2D‐EPI reference scan with the same EPI train as used for the 3D imaging readout, z‐location specific ghosting parameters could be obtained to construct a modified ACS dataset. This could lead to better reconstructions in 3D unaliasing, where a single ghost‐correction applied to the entire volume will not cover the spatial variation as reliably. It must be noted, however, that 3D‐EPI sequences are known to be affected by shot‐to‐shot phase fluctuations 34, 35 and that these variations along the kz‐encoding could well be more influential than the spatial variation of the Nyquist ghosting when it comes to reconstruction fidelity of 3D data that is undersampled in two dimensions.

## CONCLUSIONS

The presented work shows that split‐slice‐GRAPPA and 1D‐NGC‐SENSE‐GRAPPA perform all but identically in all cases. In tests where good reference ACS data were available, 2D‐NGC‐SENSE‐GRAPPA showed reduced error propagation compared to the serial 1D methods, but this relationship reversed when inferior reference data were used, stressing the importance of high quality reference data for SMS reconstruction. The proposed 2D‐NGC‐SENSE‐GRAPPA algorithm can reconstruct SMS EPI data robustly in the presence of Nyquist ghosting, and can be an attractive alternative to serial 1D methods due to its lower computational demands.

## APPENDIX 1 Computational Complexity

Calculation times for kernel inversion can be predicted provided the numerical implementation of the pseudoinverse is known. In Matlab's “pinv.m” the complexity is ∼O(*mn* ^2^), with *m*>n of the *[m x n]* matrix. The calculation time of the kernel inversion is proportional to:

$$Time\;∼\;Number\;of\;kernel\;positions\;\times \;{\left(Number\;of\;source\;points\;in\;kernel\right)}^{2}$$

with:

$$Number\;of\;source\;points\;in\;kernel\;=\;NrCoils\;\times \;K_{1}\times \;K_{2}$$

where K_i_ denotes kernel sizes in each dimensionand

$$Number\;of\;kernel\;positions\;=\left(NrLines_{1}-\;\left(K_{1}-1\right)\times AF_{1}\right)\;\times \;\left(NrLines_{2}-\;\left(K_{2}-1\right)\times AF_{2}\right)$$

where AF_i_ denotes the acceleration factor.For a large number of calibration lines, the (K‐1)×AF terms become negligible, and the number of kernel positions equates to the number of elements in the imaging matrix. Setting AF_1_ to the SMS factor, AF_2_ to the in‐plane acceleration factor AF_PE_, and M to the size of a square imaging matrix, the comparison between the calculation times can easily be interpreted. Please note that the n^2^ factor is the same in all methods when equal kernel sizes are used for fair comparison and hence can be ignored.

### Slice‐GRAPPA and 1D‐SENSE‐GRAPPA

$$\begin{array}{l} Time∼\left(SMS\times M\right)\times {\left(M/AF_{PE}\right)}^{}\times n^{2}+SMS\times M^{2}\times n^{2} \end{array}$$

where the first term reflects the slice unaliasing, and the second the in‐plane GRAPPA kernels

### 2D‐SENSE‐GRAPPA

$$Time∼\left(SMS\times M\right)\times M^{}\times n^{2}.$$

The second term in the serial 1D methods equates to that of 2D‐SENSE‐GRAPPA, meaning the latter will always be faster unless memory limitations become relevant. In Figure 6B, the predicted values for inversion (scaled to the measured value of the largest kernel) were calculated without the aforementioned (M≫K) simplification of the *n* dimension.

The complexity of the kernel *application* stage can be calculated similarly. This is dependent on the number of possible kernel positions in the target k‐space and on the number of elements in the kernel (∼O(*NrPositions × NrElements*)) but it should be noted that kernel application can be significantly accelerated by applying it in image‐space 31 and, therefore, could affect efficiency calculations.
